# Concurrent Use of Anticholinergic and Antidementia Medicines in Older Adults with Dementia: A Systematic Review of Prevalence, Predictors and Clinical Outcomes

**DOI:** 10.3390/medsci14040426

**Published:** 2026-07-24

**Authors:** Zahraa Falfaly, Gregory M. Peterson, Woldesellassie M. Bezabhe, Mohammed S. Salahudeen

**Affiliations:** School of Pharmacy and Pharmacology, Health, University of Tasmania, P.O. Box 845, Hobart, TAS 7006, Australia; zahraa.falfaly@utas.edu.au (Z.F.); g.peterson@utas.edu.au (G.M.P.); woldesellassie.bezabhe@utas.edu.au (W.M.B.)

**Keywords:** dementia, anticholinergic burden, acetylcholinesterase inhibitors, medication safety, deprescribing, prescribing cascade, older adults

## Abstract

**Background**: Concurrent use of acetylcholinesterase inhibitors (AChEIs) and medicines with anticholinergic activity is a clinically important medication safety concern in dementia care because these medicines may pharmacologically oppose dementia treatment and increase the risk of avoidable harm. This review systematically synthesised evidence on the prevalence, patterns, predictors and clinical consequences of concurrent use of anticholinergic and anti-dementia drugs (ADDs) in older adults with dementia. **Methods**: A PRISMA-informed systematic review was conducted employing Ovid MEDLINE, Embase and PsycINFO from January 2010 to April 2026. Observational studies were eligible if they included older adults with dementia and reported concurrent exposure to at least one anticholinergic medicine and an ADD, defined as AChEIs and/or memantine. Study quality was appraised using the Newcastle–Ottawa Scale. **Results**: Twenty-eight studies were included, spanning community, outpatient memory-clinic, acute hospital, long-term care and population-based settings. Two distinct exposure constructs were identified: (1) temporal co-prescribing and (2) cumulative anticholinergic burden among ADD users. Reported prevalence varied markedly according to the exposure definition and setting. Concurrent use was lowest in studies limited to bladder antimuscarinic overlap, generally around 5–13%,but increased to 20–45% when broader anticholinergic definitions were used. In long-term care and institutional cohorts, concurrent exposure reached 61–67%. Recurring predictors included polypharmacy, fragmented care or multiple prescribers, urinary symptoms, behavioural and psychological symptoms of dementia, Parkinson’s disease and non-geriatric prescribing. One large population-based study reported that high anticholinergic burden was associated with treatment modification of AChEIs (aHR 1.12), delirium (aHR 1.52), and two-year mortality (aHR 1.23). Additional studies reported associations with longer hospital stays or readmission. **Conclusions**: Concurrent use of anticholinergic medicines and ADDs is a common, clinically important and potentially modifiable medication-safety problem in dementia care.

## 1. Introduction

Dementia is a progressive neurodegenerative syndrome and a major cause of disability, dependency, and healthcare utilisation among older adults worldwide [1,2]. Pharmacological treatment remains largely symptomatic [3,4]. Acetylcholinesterase inhibitors (AChEIs), including donepezil, rivastigmine and galantamine, aim to enhance central cholinergic neurotransmission, while memantine modulates N-methyl-D-aspartate receptor activity [5,6,7]. These medicines may provide modest improvements or stabilisation in cognition, function or global clinical status for selected people with Alzheimer’s disease and related dementias, but the expected benefit is limited and can be undermined by concurrent prescribing of medicines with anticholinergic activity [8,9,10].

Anticholinergic medicines are prescribed for many indications, including urinary urgency, gastrointestinal spasm, nausea, allergy, depression, insomnia, pain, and Parkinsonian symptoms [11,12]. Their adverse effects are particularly important in older adults and people living with dementia because central and peripheral anticholinergic effects can present as confusion, delirium, constipation, urinary retention, dry mouth, blurred vision, sedation, dizziness, falls and functional decline [1,13,14,15,16,17,18,19]. Specifically, anticholinergic-induced falls are thought to result from a convergence of central effects (sedation, dizziness, cognitive impairment and slowed reflexes) and peripheral effects (blurred vision due to pupillary dilation and inability to accommodate as well as impaired balance), collectively compromising gait stability and the capacity to respond to environmental hazards [20]. The concern is amplified when anticholinergic medicines are used in people receiving AChEIs because the pharmacological actions are directionally opposed; one attempts to enhance cholinergic signalling, while the other blocks muscarinic cholinergic effects [9,21].

Clinical guidance consistently recommends caution with medicines that contribute to anticholinergic burden in older adults and those with cognitive impairment. The 2023 American Geriatrics Society Beers Criteria identify strong anticholinergic medicines as potentially inappropriate in many older adults, particularly because of the cumulative risk of falls, delirium and cognitive impairment [22]. STOPP/START version 3 includes expanded criteria to detect potentially inappropriate prescribing, including drug–disease and drug–drug interactions relevant to medicines with anticholinergic properties [23]. NICE dementia guidance similarly recommends awareness of medicines associated with increased anticholinergic burden and, where possible, minimising their use or seeking alternatives in people being assessed or treated for dementia [24].

Despite these recommendations, real-world prescribing data suggests that concurrent anticholinergic and ADD use remains frequent [25,26,27]. This may occur because of multimorbidity, fragmented prescribing across multiple clinicians, incomplete medication reconciliation, lack of visibility of over-the-counter medicines, and therapeutic uncertainty when balancing symptom control against cognitive and functional harms [28,29]. Prescribing cascades are also important. For example, urinary frequency or incontinence after initiation of an AChEI may be misinterpreted as a new condition and treated with a bladder antimuscarinic, rather than being recognised as a potential adverse effect and managed with safer alternatives [30,31].

Previous work has examined specific prescribing problems, including the concurrent use of AChEIs and bladder antimuscarinic, but the broader evidence on prevalence, predictors, medication classes and clinical consequences across care settings has not been synthesised in a way that informs medication safety practice [14,25,27,32]. This systematic review aimed to synthesise observational evidence on concurrent use of anticholinergics and ADDs in older adults with dementia. The specific objectives were to describe prevalence and patterns across settings, identify medicine classes contributing to increased anticholinergic burden, summarise patient- and system-level predictors, examine prescribing cascades, and evaluate associations with clinically important outcomes.

## 2. Methods

### 2.1. Design and Reporting Framework

This systematic review was conducted and reported in accordance with the Preferred Reporting Items for Systematic Reviews and Meta-Analyses (PRISMA) 2020 statement [33]. This study was not registered in PROSPERO.

### 2.2. Eligibility Criteria

Eligibility criteria were defined using a modified PICO framework appropriate for a descriptive medication use review. Studies were eligible if they included a cohort of older adults with documented dementia or Alzheimer’s disease and reported concurrent exposure to at least one anticholinergic medicine and at least one ADD, defined as an AChEI and/or memantine. Memantine users were included because the review aimed to capture concurrent anticholinergic exposure among older adults receiving pharmacological treatment for dementia, including both AChEIs and memantine. Database studies were also eligible if they defined concurrency through overlapping prescription or dispensing windows, such as same-day prescribing, at least 7 days of overlap, 30- to 60-day windows, or exposure within a defined period after initiation of an ADD.

Studies were included if they reported the prevalence of concurrent use and predictors, relevant medicine classes, or prescribing cascades. Clinical outcomes associated with concurrent exposure were noted, when reported. Eligible outcomes included cognitive decline, functional decline, delirium, falls, fractures, hospital length of stay, readmission, treatment modification, discontinuation of ADD, nursing-home placement and mortality.

Anticholinergic exposure within the studies could be defined using recognised burden scales or lists, including the Anticholinergic Cognitive Burden (ACB) scale, Anticholinergic Risk Scale (ARS), Anticholinergic Drug Scale (ADS), Duran list, Beers-based criteria or similar medicine lists [17,34]. Observational study designs, including cross-sectional, retrospective cohort, prospective cohort, registry-based and administrative claims studies, were included. Case reports, editorials, conference abstracts, narrative commentaries and review articles without original data were excluded. Studies focusing on anticholinergic exposure without reference to concurrent ADD use were excluded unless results were stratified for people receiving ADDs.

### 2.3. Search Strategy, Study Selection and Assessment of Quality

MEDLINE, Embase and PsycINFO were searched from 1 January 2010 to 1 April 2026. Search concepts included dementia or Alzheimer’s disease; acetylcholinesterase inhibitors, donepezil, rivastigmine, galantamine and memantine; anticholinergic or antimuscarinic medicines; anticholinergic burden; concurrent prescribing; co-prescribing; dual use; inappropriate prescribing; and polypharmacy. The full search strategy is provided in Supplementary Table S1.

All records were imported into EndNote (2025 Version; Clarivate, Philadelphia, PA, USA) and duplicates were removed before screening. Two reviewers (ZF and MS) independently screened titles and abstracts, retrieved full texts and assessed study eligibility and quality. Disagreements were resolved by discussion, with input from a third reviewer (GP) where required.

The methodological quality of the included observational studies was critically appraised using an adapted Newcastle–Ottawa Scale (NOS) [35]. The NOS was selected because all included studies were observational, including retrospective cohort, prospective cohort, cross-sectional, registry-based, administrative claims and medical-record studies. The adapted NOS assessed study quality across three broad domains: selection of the study population, comparability or adjustment for relevant confounders, and ascertainment of exposure or assessment of outcomes. A maximum of four stars could be awarded for selection, two stars for comparability and three stars for outcome/exposure assessment. Studies were classified as good, fair or poor-quality using Agency for Healthcare Research and Quality thresholds adapted for NOS interpretation [35]. A meta-analysis was not performed because of substantial heterogeneity in study design, care setting, exposure definitions, anticholinergic burden measures, follow-up periods and outcome reporting Instead, the findings were synthesised narratively and reported in accordance with the SWiM guidance where relevant [36].

### 2.4. Data Extraction and Synthesis

A standardised extraction form was used to capture study year, country, setting, design, data source, sample size, population characteristics, definition of concurrent exposure, anticholinergic burden measure, medication classes contributing to anticholinergic burden, predictors of concurrent use, prescribing cascade findings, and clinical outcomes. Definitions of exposure and outcome measures varied widely; thus, results were synthesised around five domains: study characteristics, prevalence and patterns of concurrent exposure, therapeutic classes contributing to anticholinergic burden, predictors, prescribing cascades, and clinical consequences.

## 3. Results

### 3.1. Study Selection and Characteristics

**Figure 1:**
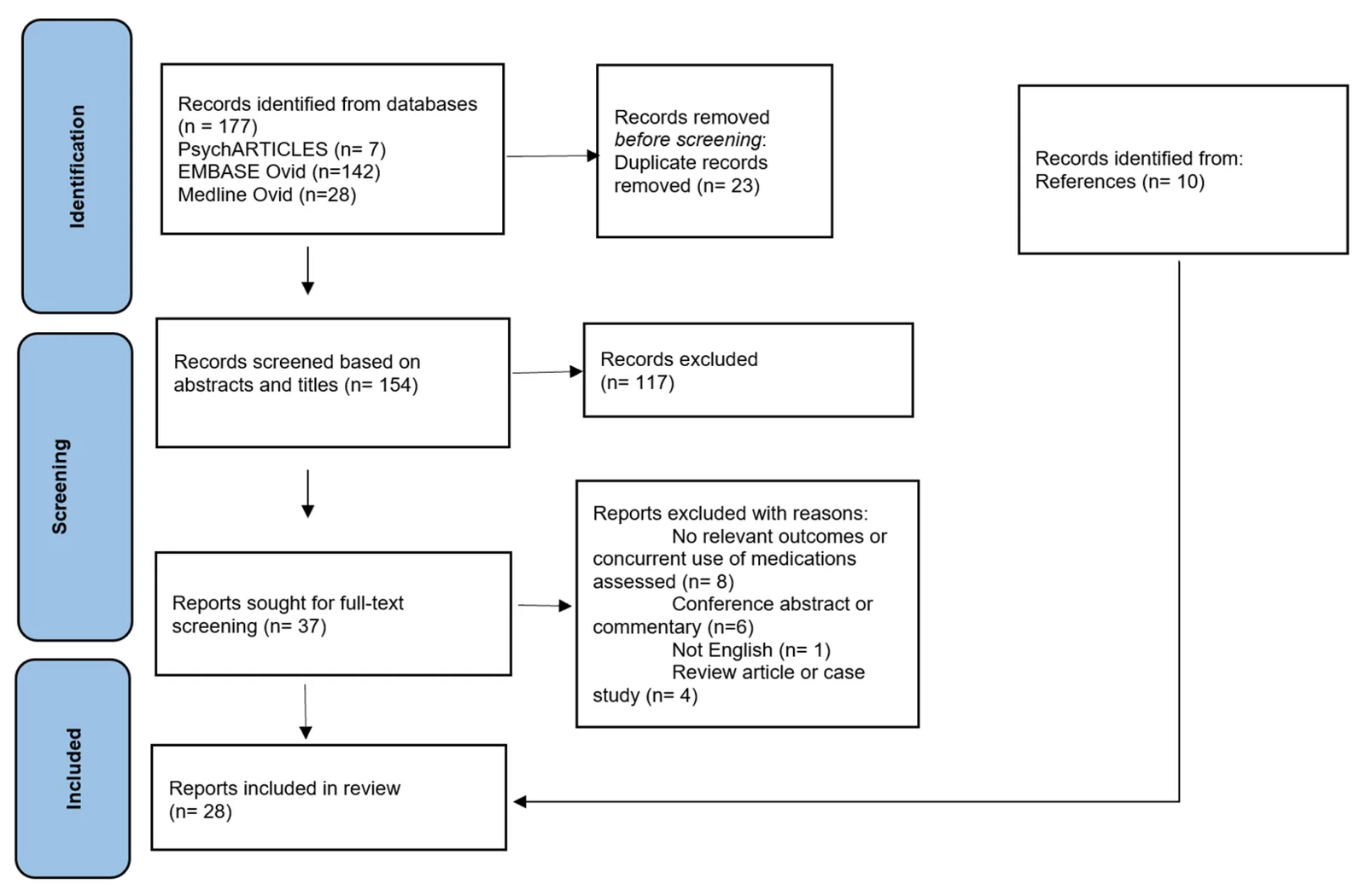
PRISMA diagram of included studies.

The search identified 177 database records, with 23 duplicates removed. After title and abstract screening, 37 reports were retrieved from the database search, and an additional 10 reports were identified through citation and reference list checking. After full-text assessment, 28 studies met the inclusion criteria and were included in the review (Figure 1).

Overall, the methodological quality of the included studies was generally acceptable. All 28 studies were rated as good quality using the adapted NOS thresholds. No study was rated as fair or poor quality. Most studies used clearly defined populations, appropriate data sources, and adequate ascertainment of exposure or outcomes. Nevertheless, common methodological limitations included heterogeneity in definitions of concurrent exposure, variation in anticholinergic burden scales, reliance on prescribing or dispensing records that may not reflect actual medicine use, and incomplete capture of over-the-counter medicines. These limitations were considered when interpreting the findings.

The 28 included studies were conducted across several geographical regions, including the United States (n = 9) [27,37,38,39,40,41,42,43,44], Canada (n = 2) [45,46], Colombia (n = 1) [47], Finland (n = 1) [32], Norway (n = 1) [9], Germany (n = 1) [48], France (n = 2) [49,50], the United Kingdom (n = 2) [51,52], South Korea (n = 3) [13,53,54], Thailand (n = 1) [10], Japan (n = 1) [55], Vietnam (n = 1) [56], and Australia (n = 3) [8,57,58]. Most studies employed retrospective cohort (n = 13) or cross-sectional (n = 11) designs and drew on administrative claims data (n = 14), electronic health records (n = 5), or national prescribing databases (n = 5), noting some studies used more than one design or data source. Sample sizes ranged from 128 patients [56] to 222,064 [37] eligible AChEI initiators, the latter from a large United States Medicare study [37].

Community and primary-care cohorts were most common settings (n = 11) [8,9,27,32,38,43,46,49,51,55,57], followed by memory-clinic outpatient cohorts (n = 4) [10,47,56,58], acute hospital settings (n = 3) [39,42,52], nursing-home or long-term care settings (n = 3) [40,44,48], and mixed population-based cohorts (n = 7) [13,37,41,45,50,53,54]. A summary of key study characteristics is included in Table 1.

### 3.2. Prevalence and Patterns of Concurrent Use

Across the 28 included studies, the reported prevalence of concurrent anticholinergic and ADD use ranged widely, from 6% to 81%, depending on the definition of anticholinergic exposure, the therapeutic classes included and the care setting [32,37,38,43,45,47,53]. When studies focused narrowly on higher burden or specific-class exposure, concurrent use was generally low to moderate, typically between 6% and 18% [32,40,44,46,48,53,57,58]. When broader definitions of any anticholinergic exposure were applied, concurrent use was moderate to high, commonly between 24% and 45% and in some populations exceeding 60%.

Concurrent use was highest in hospital settings (32% to 64%) [39,42,52] and in nursing home or long-term care populations (13% to 67%) [40,44,48], particularly when broad definitions were used. In long-term care, concurrent use of anticholinergics among people receiving AChEIs was reported in up to 61% of residents in one Canadian study [45] and 67% in a United States nursing-home analysis when broad exposure definitions were used [44]. In acute hospital cohorts, anticholinergic burden sometimes increased during admission, and discharge was identified as a key point at which psychotropic and other anticholinergic medicines may be continued or newly introduced in patients with dementia [52]. Memory-clinic outpatient cohorts showed lower rates of 8% to 12% [10,47,58], possibly reflecting more structured medication review in these specialist settings. Community and primary-care populations showed the widest variation, from 6% when only high anticholinergic burden was measured [53] to 81% when any level of anticholinergic exposure over 12 months was captured [37]. These differences underscore that the reported prevalence of concurrent use is heavily influenced by the exposure definitions and care context, and the true clinical burden may be underestimated when narrow definitions or single time points are used.

Approaches to defining anticholinergic exposure varied across studies and reflected differences in data sources, research objectives and exposure measurements. Definitions broadly fell into three categories. First, several studies used explicit temporal overlap or dispensing-window definitions, including continuous dispensing [42,56,58], at least 7 days of overlap [27], 1–2-month overlap windows [38,54,57] or 180-day pre- and post-initiation windows [8]. Second, some studies assessed exposure within fixed study windows, such as 3- or 6-month periods independent of treatment duration [9,50]. Third, several studies quantified exposure using burden or intensity metrics, including defined daily dose approaches [32,53] and scales or lists, such as ACB, ARS, ADS, Duran or Beers-based measures [40,45].

Estimates were lowest when studies focused on specific therapeutic classes, such as bladder antimuscarinics, or on clinically significant high-potency exposure. For example, studies of urinary antispasmodic or bladder antimuscarinic overlap reported prevalence estimates of 5% to 13% in some community cohorts [27,43]. In contrast, broader definitions of any anticholinergic exposure yielded substantially higher rates, often between 20% and 45% [8,48,58].

Large population-based studies showed that anticholinergic exposure around the time of dementia medicine initiation was not unusual. In a United States cohort of older adults with Alzheimer’s disease newly commencing AChEIs, 80.5% had anticholinergic exposure during the 12 months after ADD initiation when total standardised daily dose was used, although prevalence was lower at higher exposure thresholds [37]. In an Australian Pharmaceutical Benefits Scheme analysis, approximately one-third of older adults initiating dementia medicines had anticholinergic exposure before or after initiation, and almost half of post-initiation anticholinergics were prescribed by a different clinician [8].

### 3.3. Therapeutic Classes Contributing to Anticholinergic Burden

The most common contributors to anticholinergic burden were psychotropic medicines, bladder antimuscarinics, tricyclic antidepressants, sedating antihistamines and medicines used for Parkinsonian symptoms [37,53,58]. In hospital settings, psychotropics were a major driver of discharge anticholinergic burden [52], particularly antipsychotics prescribed for behavioural and psychological symptoms of dementia, delirium-related agitation, insomnia or distress [8,57]. Quetiapine, olanzapine, risperidone and tricyclic antidepressants, such as amitriptyline, were repeatedly identified as important contributors [8,45,47]. The clearest prescribing cascade involved AChEI-associated urinary symptoms with initiation of a bladder antimuscarinic [27,40,43]. Studies from Australia [8,57], Finland [32], Korea [53] and the United States [27,40] showed that urinary antispasmodics or bladder antimuscarinics may be added after initiation of an AChEI, sometimes for prolonged periods.

### 3.4. Predictors of Concurrent Use and Higher Anticholinergic Burden

Predictors were broadly consistent across studies. Polypharmacy was the most recurrent patient-level predictor, suggesting that anticholinergic exposure often reflects the cumulative consequences of managing multiple comorbidities rather than a single deliberate prescribing decision [40,45,58]. In several cohorts, each additional medicine or higher medicine count increased the likelihood of a significant anticholinergic burden [45,58].

Fragmented care was another strong system-level predictor. In Ontario, each additional five physicians involved in a patient’s care increased the odds of a higher ARS burden by 24% [45]. Australian dispensing data similarly showed that almost half of anticholinergic medicines commenced after ADD initiation were prescribed by a different clinician [8].

Other recurring predictors included urinary incontinence, behavioural and psychological symptoms of dementia [37], Parkinson’s disease [53], diabetes, younger age within the older population in some analyses [37], and female sex in several cohorts [37,45]. Care by non-geriatric prescribers was associated with higher concurrent exposure in nursing-home and claims-based studies, suggesting a protective role for geriatric expertise, specialist medication review and dementia-focused services may be associated with lower exposure [40].

### 3.5. Prescribing Cascades Following ADD Initiation

A prescribing cascade occurs when an adverse drug reaction is misinterpreted as a new condition, leading to the prescription of an additional medicine that may cause harm. A well-described cascade involves AChEIs, which may worsen or contribute to urinary symptoms through cholinergic stimulation of the detrusor muscle, potentially leading to subsequent prescribing of bladder antimuscarinics. In a cohort of patients initiating ADDs, Narayan et al. found that anticholinergic dispensing increased after ADD initiation [8]. Another Australian study found that 7% of patients who were anticholinergic-naïve in the 6 months before commencing ADDs were started on an anticholinergic afterwards [57]. Hook and colleagues reported that anticholinergic burden significantly increased during hospital admission, with 44.9% of patients taking AChEIs also receiving anticholinergic medicines during their inpatient stay [52]. Importantly, a US nursing-home study found that discontinuation of AChEIs was not consistently followed by discontinuation of anticholinergics, suggesting that once established, these cascades may become entrenched without targeted deprescribing interventions [40].

### 3.6. Clinical Outcomes Associated with Concurrent Use

Clinical outcome evidence was observational and heterogeneous [53], but several patterns emerged (Table 2). Delirium and mortality were among the most consistently reported adverse outcomes associated with higher anticholinergic burden [39,51,53]. In a large South Korean population-based study of new AChEI users, high anticholinergic burden during the first three months was associated with increased risk of treatment modification (aHR 1.12 95% CI 1.02–1.24), delirium (aHR 1.52, 95% CI 1.17–1.96) and two-year mortality (aHR 1.23, 95% CI 1.06–1.41) [53]. Anticholinergic exposure also predicted earlier AChEI discontinuation in a prospective French cohort (aHR 4.26) [49] and shorter treatment duration in a Norwegian pharmacoepidemiological study [9].

High anticholinergic burden was associated with a longer hospital length of stay in a United States inpatient cohort (median 5.5 days vs. 4.25 days in lower-burden patients) [39] and two-year mortality in the South Korean study noted above [53]. However, both associations are likely subject to residual confounding by frailty, illness severity and comorbidity, which are not fully captured in the studies.

Cognitive outcomes were the most heterogeneous. Some studies reported greater cognitive decline or lower Mini-Mental State Examination score among those with higher anticholinergic exposure [27,55], whereas others found no independent association after adjustment for baseline cognition, comorbidity or disease severity [10,38,58]. This inconsistency is not surprising, as cognitive trajectories in dementia are influenced by disease subtype, baseline severity, frailty, delirium, depression, education and care environment [53]. Nonetheless, the inconsistent findings regarding cognitive decline should not be interpreted as evidence of safety because anticholinergic exposure was independently associated with delirium, functional outcomes and hospital outcomes across multiple studies [27,39,53].

## 4. Discussion

This systematic review shows that concurrent anticholinergic and ADD use is a persistent medication-safety problem in dementia care [37,41]. Across 28 observational studies and diverse healthcare systems, concurrent exposure was common in community, hospital, memory-clinic and long-term care settings [37,42,59]. Although prevalence estimates varied widely, in part because of different exposure definitions [60], the direction of evidence was consistent: people with dementia often remain exposed to medicines that can oppose or diminish the intended effects of anti-dementia therapy and increase risk of avoidable harm [37,41]. In Parkinson’s disease, anticholinergic medicines may be used to reduce tremor by modulating the relative cholinergic–dopaminergic imbalance in the basal ganglia [41,54]. However, in patients who also have dementia or are receiving AChEIs, concurrent anticholinergic therapy creates pharmacological opposition and may increase the risk of cognitive and other anticholinergic adverse effects [41,54].

The review also highlights that concurrent use is rarely a simple single prescriber error [26]. It is usually a system problem produced by polypharmacy, multimorbidity, fragmented care, inconsistent communication across prescribers, incomplete medication histories and insufficient attention to cumulative anticholinergic burden [11,26,37]. This is clinically important because people with dementia commonly receive treatment from multiple clinicians and may have limited capacity to communicate treatment history or adverse effects [45]. It also shifts the solution from individual education alone to broader medication-safety systems [61,62]. Education can raise awareness, but safer prescribing requires reliable processes that make the cumulative anticholinergic burden visible at the point of prescribing, dispensing, medication reconciliation and discharge [61,62,63].

The findings align with established evidence that anticholinergic exposure is associated with cognitive and functional harm in older people [61,63,64]. They also extend prior work by focusing specifically on people receiving ADD, in whom the pharmacological antagonism is particularly relevant. A previous systematic review examined dual use of AChEIs and urinary anticholinergics, finding mixed cognitive and functional outcomes due to the heterogeneity of the studies and small samples in some case [60]. Cognitive decline in dementia is difficult to attribute because of confounding by disease severity, delirium, frailty, depression, survival bias and variable cognitive assessment tools. The present review was broader, incorporating anticholinergic burden across multiple medicine classes and settings, and synthesising predictors, prescribing cascades and outcomes beyond bladder antimuscarinic use alone.

The repeated association between polypharmacy and anticholinergic burden deserves careful interpretation [37,62]. Polypharmacy is not inherently inappropriate, and many people with dementia have legitimate indications for multiple medicines [11,37,42]. However, higher medicine counts increase the opportunity for exposure to low-, moderate- and high-potency anticholinergic medicines [37,62]. Polypharmacy therefore acts as a useful marker for medication-review priority rather than a standalone indicator that treatment is inappropriate [61,62]. In practice, the question should not be simply how many medicines a person takes, but whether each medicine has a clear indication, measurable benefit, acceptable risk and ongoing alignment with patient and caregiver goals [63,64].

Several clinical implications follow from the evidence. First, anticholinergic burden should be assessed before ADDs are initiated and re-evaluated whenever new urinary, sleep, behavioural or gastrointestinal symptoms emerge. New symptoms should be investigated as possible adverse effects or prescribing cascades before additional medicines are commenced [26,37,60]. Pharmacist-led medication review with feedback to prescribers has been among the most extensively studied interventions for reducing anticholinergic burden in older adults [61,62] and pharmacist-led deprescribing using population health management tools has been shown to be feasible in older adults with dementia [65]. These reviews are especially valuable at hospital admission, discharge, memory-clinic assessment, residential aged-care entry and annual medication review, where a pharmacist can reconcile medications across care boundaries and identify cumulative anticholinergic exposure that might otherwise go unrecognised [66,67].

Electronic health records and prescribing systems should make cumulative anticholinergic burden visible at the point of prescribing. Digital tools for anticholinergic risk calculation are increasingly available and can be integrated into clinical workflows [68,69], and alerts should be linked to practical alternatives rather than simply warning clinicians of the problem [65]. Future research should also improve exposure measurement. Many anticholinergic burden scales use point estimates and do not fully account for dose, duration, adherence, blood–brain barrier penetration, drug-specific pharmacology or over-the-counter medicines [60,63,69]. Dose- and duration-adjusted approaches, such as total standardised daily dose, may better reflect cumulative exposure, but require validation against clinical outcomes in dementia populations [37].

This review has several strengths. It used a systematic search across multiple databases, incorporated citation checking, focused on a clinically important pharmacological interaction and synthesised evidence across community, hospital, memory-clinic and long-term care settings. It also considered medication classes, predictors, prescribing cascades and outcomes, which provides a practice-oriented overview rather than a prevalence estimate alone.

Some limitations are important to note. First, all included studies were observational, so causal inference is limited and confounding by indication, dementia severity, frailty and comorbidity is likely. Second, definitions of concurrent exposure varied widely, from same-day prescribing to multi-month windows and cumulative burden scores, which prevented valid pooling. Third, many studies used dispensing or prescribing data, which may not reflect actual medicine-taking and often miss over-the-counter medicines.

Cognitive outcomes were measured inconsistently and may have been affected by baseline disease severity, delirium, attrition and survivor bias. Of note, the ACB scale includes the use of inhaled anticholinergics and does not differentiate drugs based on blood–brain barrier permeability, although bladder antimuscarinics can vary considerably in this property [27]. Blood–brain barrier permeability may increase with age and neurodegenerative disease, meaning that the current scales may misestimate anticholinergic risk [32,52]. Future anticholinergic burden tools should incorporate blood-brain permeability data to provide more precise risk stratification. Finally, the review was restricted to English-language literature, which may have excluded relevant regional evidence.

## 5. Conclusions

Concurrent use of anticholinergic medicines and ADDs is common in older adults with dementia and represents a clinically important form of potentially avoidable pharmacological antagonism. The available observational evidence suggests that concurrent use may be associated with adverse clinical outcomes, including delirium, functional decline, treatment modification, hospital-related outcomes and mortality, although these findings should be interpreted in the context of residual confounding and heterogeneity across studies. Routine assessment of anticholinergic burden at the time of ADD initiation, during medication reconciliation, and across transitions of care may provide a practical system-level strategy to identify and reduce avoidable anticholinergic exposure. Health systems should support coordinated medication review, electronic prescribing alerts and deprescribing pathways that reduce unnecessary anticholinergic burden while maintaining symptom control, function and quality of life in people living with dementia.

## Figures and Tables

**Table 1:** Characteristics of included studies; characterised based on setting and further stratified by burden definition or overlap of use.

Study (Year) Country	Design/Data Source	Population	Concurrent Use Definition	Key Findings	Interpretation/Relevance to This Review
Community/Outpatient Setting					
Overlap-Based Concurrent Use Definition					
Boudreau et al. (2011) USA (Washington & Colorado) [38]	Retrospective cohort; Group Health/Kaiser data, 2000–2007.	5625 people initiated AChEIs; community-based cohort, mean age approximately 78 years.	At least one moderate-to-high potency anticholinergic medicine overlapping with AChEI therapy.	Thirty-seven percent of AChEI users had at least one concurrent anticholinergic medicine; 11% had two or more. Median overlap was about 4 months, and 25% had overlap for more than 12 months. No significant adjusted association was found with 2-year mortality or nursing-home placement.	Demonstrated that concurrent exposure is common and can be prolonged, although some long-term outcomes may be difficult to detect after adjustment.
Torvinen-Kiiskinen et al. (2014) Finland [32]	Register-based cohort; MEDALZ data, 2006–2009.	20,442 community-dwelling people with Alzheimer’s disease using AChEIs.	Overlap between AChEI and urinary antispasmodic.	Concomitant urinary antispasmodic use occurred in 7.3% of AChEI users. Median overlap was 236 days, and 39% had overlap for at least one year. Risk factors included age < 80 years, male sex and Parkinson’s disease.	Provided clear evidence of prolonged AChEI–urinary antispasmodic overlap.
Gadzhanova et al. (2015) Australia [57]	Retrospective cohort; Pharmaceutical Benefits Scheme claims, 2009–2010.	24,110 people aged ≥65 years newly dispensed an AChEI or memantine.	Anticholinergic or sedative medicine dispensed before and after initiation of ADDs using ARS/ADS-related measures.	Anticholinergic/sedative medicine use increased after initiation of dementia medicines. Approximately 18% had concurrent anticholinergic exposure after initiation.	Supported the need to review existing anticholinergic and sedative medicines when ADDs are commenced.
Green et al. (2020) United States [27]	Retrospective cohort: National Health and Aging Trends Study linked with Medicare claims, 2011–2014.	698 people with probable dementia; community-based Medicare cohort.	Overlap of bladder antimuscarinic and AChEI treatment for at least 7 days; anticholinergic burden measured using ACB.	Thirty-two percent used bladder antimuscarinics concurrently with AChEIs. Falls and delirium were highlighted as important signals for medication review.	Provides focused evidence on the AChEI–bladder antimuscarinic prescribing cascade.
Green et al. (2017) United States [43]	Cross-sectional analysis; NACC Uniform Data Set, 2005–2015.	24,106 older adults, including cognitively normal participants, people with MCI and people with dementia.	Anticholinergic Drug Scale score ≥ 2, including bladder antimuscarinic exposure.	Antimuscarinic prescribing was common among older adults with cognitive impairment; concurrent AChEI and antimuscarinic use was reported in 41% of relevant users.	Reinforced that bladder antimuscarinic use remains common despite cognitive safety concerns.
Jenraumjit et al. (2020) Thailand [10]	Retrospective longitudinal cohort with 1-year follow-up.	133 older adults with Alzheimer’s disease receiving AChEIs in outpatient care.	Anticholinergic burden defined as ACB score ≥ 1.	Anticholinergic medicines were used by 31.6% of patients. Anticholinergic exposure and older age were associated with poorer long-term cognitive outcomes.	Provided outcome evidence linking anticholinergic exposure with cognitive decline in AChEI-treated patients.
Kurata et al. (2015) Japan [55]	Retrospective prescription survey; electronic medication records from community pharmacies, 2007–2012.	431 outpatients prescribed donepezil; community pharmacy setting.	Concurrent donepezil and medicines with ACB score ≥ 1.	Concurrent anticholinergic prescribing occurred in 24.1% of donepezil users. Among concurrent users, 19.2% had cumulative ACB score > 4.	Demonstrated that community pharmacy records can identify clinically important anticholinergic exposure among donepezil users.
Valladales-Restrepo et al. (2019) Colombia [47]	Cross-sectional study, 2018.	4134 Alzheimer’s disease patients receiving AChEIs or memantine.	Any anticholinergic medicine according to ACB scale.	Concurrent use occurred in 7.8% of patients. Age > 85 years was associated with high anticholinergic burden.	Adds evidence from a middle-income setting and supports age-related vulnerability.
Gardette et al. (2010) France [49]	Prospective multicentre cohort; REAL.FR study.	611 community-dwelling patients with mild-to-moderate Alzheimer’s disease receiving AChEIs.	Self-reported concurrent anticholinergic and AChEI use at study visits.	6.1% of Alzheimer’s dementia patients were on an anticholinergic. Anticholinergic use was associated with AChEI discontinuation; reported aHR for discontinuation was 4.26.	Supported the need to review existing anticholinergic and sedative medicines when anti-dementia therapy is commenced.
**Studies using burden scale to assess concurrency**					
Zhu et al. (2019) Canada [46]	Cross-sectional analysis; linked administrative health data.	36,845 older adults with dementia taking AChEIs.	Use of at least one medicine with high anticholinergic burden with ADD on index date.	High anticholinergic burden medicines were used by 10.6% of patients receiving AChEIs. Rates were similar in females and males. Low burden not assessed.	Showed that high-burden anticholinergic exposure was common among AChEI users at population level.
Reppas-Rindlisbacher et al. (2016) Ontario, Canada [45]	Population-based cross-sectional study; linked Ontario administrative health databases.	79,067 community-dwelling adults and 12,113 long-term care residents aged ≥66 years initiating AChEIs.	Anticholinergic Risk Scale score ≥ 2 in the year before AChEI initiation.	Anticholinergic burden was present in 27% of community-dwelling AChEI initiators and 61% of long-term care residents. Seventy-seven percent of anticholinergic medicines were not discontinued after AChEI initiation. Each additional five physicians increased the odds of higher burden.	Provided strong evidence that fragmented care and multiple prescribers contribute to persistent burden.
Ah et al. (2019) South Korea [53]	Retrospective cohort; National Health Insurance Senior Cohort, 2003–2011.	25,825 new AChEI users with dementia aged ≥65 years; population-based cohort.	High anticholinergic burden defined as ACB score > 3 during the first 3 months of AChEI therapy.	High anticholinergic burden occurred in 6.0% of patients. It was associated with treatment modification, delirium and mortality; reported estimates included aHR 1.12 for treatment modification, aHR 1.52 for delirium and aHR 1.23 for mortality.	Provided important evidence that early high anticholinergic burden after AChEI initiation is associated with clinically meaningful harm.
Talwar et al. (2024) United States [37]	Retrospective longitudinal cohort; claims data, 2013–2017.	222,064 community-dwelling older adults with Alzheimer’s disease newly initiating AChEIs.	Total standardised daily anticholinergic exposure over 12 months.	Any anticholinergic exposure occurred in 80.5% of patients. Exposure was moderate in 36.3% and high in 24.8%. Baseline burden was the strongest predictor of post-initiation burden.	Provided the highest population-level estimate and showed the importance of reviewing anticholinergic medicines before AChEI initiation.
Montastruc et al. (2015) France [50]	Cross-sectional study; French Pharmacovigilance Database.	475 patients with Alzheimer’s disease.	Anticholinergic exposure assessed using ADS and Duran list.	Anticholinergic burden was identified in 59.4% using ADS and 45.1% using the Duran list. Clinically significant burden was present in 18.2%.	Showed that prevalence varies substantially depending on the anticholinergic scale used.
**Long-term care/nursing-home setting**					
**Overlap-based concurrent use definition**					
Gromek et al. (2023) United States [40]	Retrospective analysis; linked Medicare and nursing-home data, 2015–2016.	37,087 nursing-home residents aged ≥65 years with severe dementia receiving AChEIs.	Overlap of strong anticholinergic medicines, according to AGS Beers Criteria, within the first 14 days of nursing-home stay.	Fifteen percent had anticholinergic co-prescribing. Higher odds were associated with female sex, polypharmacy and non-geriatrician prescribers. Discontinuation of AChEIs influenced subsequent anticholinergic deprescribing patterns.	Highlighted nursing homes as a high-risk setting and supported geriatrician/pharmacist involvement in deprescribing.
Palmer et al. (2014) USA [44]	Retrospective analysis; Medicare linked with Minimum Data Set.	69,877 nursing-home residents aged ≥65 years with dementia.	Concomitant use of AChEIs with medicines having ACB score 2 or 3. Based on the Indiana Drug Discovery Network Anticholinergic Burden Scale.	Among AChEI users, the average monthly usage of an anticholinergic was 81%.	Supported residential aged care as a priority setting for deprescribing high-risk anticholinergics.
**Hospital/inpatient setting**					
**Overlap-based concurrent use definition**					
Schultz et al. (2017) United States [42]	Retrospective electronic health record review, 2006–2010.	304 hospitalised patients receiving ADDs.	ADD and anticholinergic medicine prescribed during the same hospital encounter.	Most concurrent prescriptions involved AChEIs rather than memantine. Nine percent of patients receiving dementia medications had concurrent use. High-potency anticholinergics accounted for 64.1% of anticholinergic exposure.	Highlighted clinically relevant concurrent prescribing in hospitalised patients. Lower rates in Asian patients.
**Studies using burden scale to assess concurrency**					
Hook et al. (2022) United Kingdom [52]	Multicentre cross-sectional audit.	352 hospital inpatients with a formal dementia diagnosis who were ready for discharge.	Anticholinergic Effect on Cognition scale; comparison of burden at admission and discharge.	Mean anticholinergic burden increased from 0.61 at admission to 0.77 at discharge. Among patients receiving AChEIs, 44.9% also received anticholinergic medicines. Psychotropics contributed substantially to the increase.	Indicated that hospitalisation may increase anticholinergic burden, particularly through psychotropic prescribing.
Guan et al. (2024) United States [39]	Retrospective cohort; electronic medical-record review, 2022–2023.	237 hospitalised older adults receiving donepezil or rivastigmine.	Anticholinergic Cognitive Burden scale; high burden defined as ACB ≥ 3.	44.9% of patients taking AChEIs were taking an anticholinergic. High anticholinergic burden was associated with longer hospital length of stay and higher 30-day readmission. Median length of stay was 5.5 days in the high-burden group versus 4.25 days in the lower-burden group.	Supported hospital admission and discharge as key points for anticholinergic burden review.
**Population-based/National register studies**					
**Overlap-based concurrent use definition**					
Baik et al. 2022 South Korea. [54]	Cross-sectional analysis of HIRA-NAPS.	8394 PD patients with dementia. The total number in the dataset was approximately 700,000 older patients.	Overlap of ≥2 months between anticholinergic medicines for Parkinson’s disease motor symptoms and AChEIs.	Concurrent use occurred in 9.6% of patients. Mean overlap was approximately 7.7 months, and one-third of co-prescribed patients used both medicine classes throughout the year. Most co-prescribing was from the same prescriber.	Showed that concurrent prescribing may be intentional or accepted in some clinical contexts, particularly Parkinson’s disease, but still creates pharmacological opposition.
Mantri et al. (2019) United States [41]	Cross-sectional analysis; Medicare claims, 2014.	268,407 Medicare beneficiaries with Parkinson’s disease.	Overlap of high-potency anticholinergic medicines with AChEIs.	Among AChEI users, 44.5% experienced at least one high-potency anticholinergic–AChEI overlap event. Women and Hispanic beneficiaries had higher odds of concurrent exposure.	Highlighted potentially inappropriate prescribing in Parkinson’s disease dementia and suggested demographic variation in exposure.
Narayan et al. (2019) Australia [8]	Pharmacoepidemiological cohort; PBS 10% random sample, 2013–2017.	4393 older adults initiating ADDs.	Any dispensing of an anticholinergic medicine within 180 days before or after the dementia medicine initiation.	Approximately one-third of people were exposed to anticholinergic medicines before or after initiation. Forty-six percent continued the same anticholinergic medicine before and after initiation, and forty-nine percent of post-initiation anticholinergics were prescribed by a different clinician.	Identified fragmented care as a driver of persistent anticholinergic exposure.
Nguyen et al. (2018) Vietnam [56]	Retrospective medical-record review, 2015–2016.	128 dementia outpatients.	AChEI and anticholinergic medicine prescribed at the same hospital visit.	Approximately 43.5% of patients were prescribed an anticholinergic medicine at the same visit as an AChEI.	Suggested high concurrent exposure in specialist outpatient care and the need for coordinated medication review.
Sverdup Efjestad et al. (2017) Norway [9]	Prospective pharmacoepidemiological study; Norwegian Prescription Database, 2005–2011.	17,321 AChEI users aged 65–80 years; detailed analysis of persistent users.	ADS score calculated for medicines prescribed during the first 6 months of AChEI therapy.	Anticholinergic comedication was associated with shorter AChEI treatment duration. Antipsychotic co-prescribing was strongly associated with early AChEI discontinuation.	Suggests that unfavourable comedications may reduce persistence with dementia treatment.
**Studies using burden scale to assess concurrency**					
Joung et al. (2020) South Korea [13]	Population-based cohort; National Health Insurance Service data, 2012.	388,629 adults aged ≥70 years, including 9547 with Alzheimer’s disease.	Heavy anticholinergic use, defined as ≥90 standardised prescribed doses in one year.	Heavy anticholinergic use was more common in people with Alzheimer’s disease than those without it, 22.5% versus 16.3%. Low ACB score medications not assessed.	Showed that people with Alzheimer’s disease are disproportionately exposed to substantial anticholinergic burden.
Scheel-Barteit et al. (2025) Germany [48]	Longitudinal multicentre open-registry study; baseline cross-sectional analysis.	96 people with dementia from a nursing-care registry.	Regular anticholinergic medication identified using the German ACB scale.	Any anticholinergic exposure was present in 65.6% of patients; clinically relevant burden, defined as ACB ≥ 3, occurred in 13.5%.	Confirmed high anticholinergic exposure in nursing-care populations and the need for regular review.
**Mixed/multiple settings**					
**Studies using burden scale to assess concurrency**					
Barry et al. (2016) Northern Ireland, UK [51]	Retrospective cross-sectional study; Enhanced Prescribing Database.	6826 community-dwelling people with dementia receiving at least one dementia medicine.	Potentially inappropriate prescribing according to STOPP/Beers-related criteria, including anticholinergic medicines.	Potentially inappropriate prescribing occurred in 64.4% of patients. Anticholinergic medicines were the most common potentially inappropriate prescribing category, affecting 25.2%. Polypharmacy strongly increased risk.	Highlighted the contribution of anticholinergic medicines to inappropriate prescribing in community dementia care.
Cross et al. (2016) Australia [58]	Cross-sectional baseline analysis; PRIME memory-clinic cohort.	964 community-dwelling patients attending memory clinics with mild cognitive impairment or dementia.	Clinically significant anticholinergic burden defined as ACB score ≥ 3; PIMs assessed using Beers and STOPP criteria.	44.9% of AChEI users also used an anticholinergic. Polypharmacy and lower education were associated with higher burden.	Suggested that memory-clinic medication review should include formal anticholinergic burden assessment.

Abbreviations: ACB, anticholinergic burden; ADD, anti-dementia drugs; AChEI, acetylcholinesterase inhibitor; ADS, Anticholinergic Drug Scale; aHR, adjusted hazard ratio; ARS, Anticholinergic Risk Scale; PD, Parkinson’s disease; PIM, potentially inappropriate medicine.

**Table 2:** Summary of key clinical outcomes associated with concurrent anticholinergic and ADD use.

Outcome	Anticholinergic Exposure Metric	Summary of Findings	Interpretation
Delirium	High ACB burden or ACB score > 3 in early exposure windows	High burden was associated with increased delirium risk in population-based analyses; one estimate reported aHR 1.52 (95% CI 1.17–1.96) [53].	One of the strongest and most clinically plausible outcome signals.
Mortality	High ACB burden in first months after AChEI initiation	Higher two-year mortality was reported in one large cohort (aHR 1.23, 95% CI 1.06–1.41) [53].	Suggestive association; residual confounding by frailty and illness severity remained likely.
Treatment modification or discontinuation	Anticholinergic exposure at baseline or high ACB burden	Anticholinergic use predicted switching or discontinuation of AChEIs [49] in prospective and claims-based cohorts; aHR 1.12 95% CI 1.02–1.24 [53]. Treatment discontinuation was earlier in those with a higher anticholinergic burden than those with lower score [9].	Supported the idea that concurrent exposure may undermine persistence with dementia therapy.
Falls and fracture	Any anticholinergic use or high anticholinergic exposure.	Some studies reported increased falls and fractures with anticholinergic exposure although not all adjusted analyses were statistically significant [27,37].	Clinically important outcome; influenced by frailty, psychotropic exposure and mobility.
Cognitive decline	Any anticholinergic exposure, high baseline ACB, cumulative burden.	Findings varied. Some studies reported greater decline in cognitive scores [10,53], whereas others found no independent association after adjustment [10].	Mixed evidence; cognitive outcomes were difficult to isolate in observational dementia cohorts.
Hospital outcomes	High ACB burden during admission or at discharge.	High burden was associated with longer hospital length of stay [27,39] in an inpatient cohort. Readmission and nursing home admission findings were less consistent [38,39].	Hospitalisation and discharge are key medication-review opportunities.

Abbreviations: ACB, anticholinergic burden score; aHR, adjusted hazard ratio; CI, confidence interval.

## Data Availability

No new data were created or analyzed in this study. Data sharing is not applicable to this article.

## Supplementary Materials

The following supporting information can be downloaded at https://www.mdpi.com/article/10.3390/medsci14040426/s1, Table S1: Medline Search Strategy; Table S2: The Newcastle-Ottawa scale risk of bias assessment for included cohort and case-control studies.

## Abbreviations

The following abbreviations are used in this manuscript:

ACB	Anticholinergic Burden Score
ADD	Anti-Dementia Drug
AChEI	Acetylcholinesterase Inhibitor
ADS	Anticholinergic Drug Scale
ARS	Anticholinergic Risk Scale
