# Supplementary material for: Concurrent Use of Anticholinergic and Antidementia Medicines in Older Adults with Dementia: A Systematic Review of Prevalence, Predictors and Clinical Outcomes
Source: Med Sci (Basel). 2026 Jul 24;14(4):426. doi: 10.3390/medsci14040426 (PMC13515873; doi:10.3390/medsci14040426)
Supplement: Supplementary file 1 [file medsci-14-00426-s001.zip › medsci-4423567-supplementary.pdf]

Table S1. Medline search strategy

Source	Search strategy: keyword/MeSH
MEDLINE (Ovid SP) January 2010 to April 2026	1. A. Dementia population<br>exp Dementia/<br>or exp Alzheimer Disease/<br>or exp Dementia, Vascular/<br>or exp Frontotemporal Dementia/<br>or Lewy Body Dementia.mp.<br>2. B. Antidementia medicines<br>exp Cholinesterase Inhibitors<br>or exp Memantine/<br>or acetylcholinesterase inhibitor*.mp.<br>or cholinesterase inhibitor*.mp.<br>or donepezil.mp.<br>or rivastigmine.mp.<br>or galantamine.mp.<br>or memantine.mp.<br>C. Anticholinergic medicines<br>3. exp Cholinergic Antagonists/<br>or exp Antimuscarinics/<br>or anticholinergic*.mp.<br>or antimuscarinic*.mp.

or anticholinergic burden.mp.

or anticholinergic drug burden.mp.

D. Combine dementia, antidementia, and anticholinergic concepts

4. 1 and 2 and 3

E. Concurrency / co-prescribing terms

5. (concurrent* or concomitant* or co-prescrib* or coadminist*

or "dual use" or "polypharmacy" or "inappropriate prescribing").mp.

F. Combine with concurrency terms

6. 4 and 5

G. Apply limits

7. limit 6 to (English language and humans and yr="2010 -Current")

H. Further limit by population

8. limit 7 to (Aged 65 and over or aged.mp. or older adults.mp.

or elderly.mp. or geriatric*.mp. or older people.mp.)

Table S2. The Newcastle-Ottawa scale risk of bias assessment for included cohort studies

Author, Year, Country	Study design	Selection	Comparability	Outcome / Exposure	Overall quality
Ah et al. (2019), South Korea [53]	Retrospective cohort	✯✯✯✯	✯✯	✯✯✯	Good
Baik et al. (2022), South Korea [54]	Cross-sectional claims analysis	✯✯✯	✯	✯✯✯	Good
Boudreau et al. (2011), USA [38]	Retrospective cohort	✯✯✯✯	✯✯	✯✯✯	Good
Barry et al. (2016), Northern Ireland, UK [51]	Retrospective cross-sectional study	✯✯✯	✯✯	✯✯✯	Good
Cross et al. (2016), Australia [58]	Cross-sectional baseline analysis	✯✯✯	✯✯	✯✯✯	Good
Gadzhanova et al. (2015), Australia [57]	Retrospective cohort	✯✯✯✯	✯✯	✯✯✯	Good
Gardette et al. (2010), France [49]	Prospective multicentre cohort	✯✯✯✯	✯✯	✯✯✯	Good
Green et al. (2020), United States [27]	Retrospective descriptive cohort	✯✯✯✯	✯✯	✯✯✯	Good
Green et al. (2017), United States [43]	Cross-sectional analysis	✯✯✯✯	✯✯	✯✯✯	Good
Gromek et al. (2023), United States [40]	Retrospective claims/MDS analysis	✯✯✯	✯✯	✯✯✯	Good
Guan et al. (2024), United States [39]	Retrospective cohort	✯✯✯✯	✯✯	✯✯	Good

Author, Year, Country	Study design	Selection	Comparability	Outcome / Exposure	Overall quality
Hook et al. (2022), United Kingdom [52]	Multicentre cross-sectional audit	☆☆☆	☆☆	☆☆☆	Good
Joung et al. (2020), South Korea [13]	Population-based cohort/cross-sectional claims study	☆☆☆☆	☆☆	☆☆☆	Good
Jenraumjit et al. (2020), Thailand [10]	Retrospective longitudinal cohort	☆☆☆	☆☆	☆☆☆	Good
Kurata et al. (2015), Japan [55]	Retrospective prescription survey	☆☆☆	☆	☆☆☆	Good
Mantri et al. (2019), United States [41]	Cross-sectional Medicare claims analysis	☆☆☆☆	☆☆	☆☆☆	Good
Montastruc et al. (2015), France [50]	Cross-sectional baseline analysis of REAL.FR cohort	☆☆☆	☆☆	☆☆☆	Good
Narayan et al. (2019), Australia [8]	Pharmacoepidemiological cohort	☆☆☆☆	☆☆	☆☆☆	Good
Nguyen et al. (2018), Vietnam [56]	Retrospective medical-record review	☆☆☆	☆	☆☆	Good
Palmer et al. (2014), USA [44]	Retrospective Medicare/MDS analysis	☆☆☆☆	☆☆	☆☆☆	Good
Schultz et al. (2017), United States [42]	Retrospective electronic health-record review	☆☆☆	☆	☆☆	Good
Reppas-Rindlisbacher et al. (2016), Canada [45]	Population-based cross-sectional study	☆☆☆☆	☆☆	☆☆☆	Good
Scheel-Barteit et al. (2025), Germany [48]	Longitudinal registry; baseline cross-sectional analysis	☆☆☆	☆☆	☆☆	Good

Author, Year, Country	Study design	Selection	Comparability	Outcome / Exposure	Overall quality
Sverdup Efjestad et al. (2017), Norway [9]	Prospective pharmacoepidemiological prescription-database study	✯✯✯✯	✯	✯✯✯	Good
Talwar et al. (2024), United States [37]	Retrospective longitudinal cohort; comparative interrupted time-series	✯✯✯✯	✯✯	✯✯✯	Good
Torvinen-Kiiskinen et al. (2014), Finland [32]	Register-based cohort	✯✯✯✯	✯✯	✯✯✯	Good
Valladales-Restrepo et al. (2019), Colombia [47]	Cross-sectional population-database study	✯✯✯	✯	✯✯	Good
Zhu et al. (2019), Canada [46]	Population-based cross-sectional study	✯✯✯✯	✯✯	✯✯	Good

<u>Note</u>: A study can be given a maximum of one star for each numbered item within the *Selection* (maximum of 4 stars)) and *Outcome* categories (maximum of 3 stars). A maximum of two stars can be given for *Comparability.*

Thresholds for converting the Newcastle-Ottawa scale to Agency for Healthcare Research and Quality (AHRQ) standards (good, fair, and poor):

*Good quality***:** 3 or 4 stars in selection domain AND 1 or 2 stars in comparability domain AND 2 or 3 stars in outcome/exposure domain

*Fair quality***:** 2 stars in selection domain AND 1 or 2 stars in comparability domain AND 2 or 3 stars in outcome/exposure domain

*Poor quality***:** 0 or 1 star in selection domain OR 0 stars in comparability domain OR 0 or 1 stars in outcome/exposure domain.
